# Photographic identification of individuals of a free‐ranging, small terrestrial vertebrate

**DOI:** 10.1002/ece3.1883

**Published:** 2016-01-18

**Authors:** Claire E. Treilibs, Chris R. Pavey, Mark N. Hutchinson, C. Michael Bull

**Affiliations:** ^1^ School of Biological SciencesFlinders UniversityGPO Box 2100AdelaideSouth Australia5001Australia; ^2^ Flora and Fauna DivisionNT Department of Land Resource ManagementPO Box 1120Alice SpringsNorthern Territory0871Australia; ^3^ CSIRO Land and Water FlagshipPO Box 2111Alice SpringsNorthern Territory0871Australia; ^4^ South Australian MuseumNorth TerraceAdelaideSouth Australia5000Australia

**Keywords:** Abundance, capture‐recapture, lizard, natural markings, non‐invasive

## Abstract

Recognition of individuals within an animal population is central to a range of estimates about population structure and dynamics. However, traditional methods of distinguishing individuals, by some form of physical marking, often rely on capture and handling which may affect aspects of normal behavior. Photographic identification has been used as a less‐invasive alternative, but limitations in both manual and computer‐automated recognition of individuals are particularly problematic for smaller taxa (<500 g). In this study, we explored the use of photographic identification for individuals of a free‐ranging, small terrestrial reptile using (a) independent observers, and (b) automated matching with the Interactive Individual Identification System (I^3^S Pattern) computer algorithm. We tested the technique on individuals of an Australian skink in the *Egernia* group, Slater's skink *Liopholis slateri*, whose natural history and varied scale markings make it a potentially suitable candidate for photo‐identification. From ‘photographic captures’ of skink head profiles, we designed a multi‐choice key based on alternate character states and tested the abilities of observers — with or without experience in wildlife survey — to identify individuals using categorized test photos. We also used the I^3^S Pattern algorithm to match the same set of test photos against a database of 30 individuals. Experienced observers identified a significantly higher proportion of photos correctly (74%) than those with no experience (63%) while the I^3^S software correctly matched 67% as the first ranked match and 83% of images in the top five ranks. This study is one of the first to investigate photo identification with a free‐ranging small vertebrate. The method demonstrated here has the potential to be applied to the developing field of camera‐traps for wildlife survey and thus a wide range of survey and monitoring applications.

## Introduction

Recognition of individuals within an animal population is central to a range of estimates about population structure and dynamics. Estimates of population density and abundance rely on an ability to distinguish individual animals, and estimates of life history parameters, such as growth rate and survival, require tracking those individuals through space and time. However, traditional methods of marking individuals, such as toe‐clipping, may cause stress, injury or infection to the animal (Reisser et al. 2008; Sacchi et al. 2010) and are ethically questionable. Capture and handling, often required to apply marking, may also affect normal behavior of an individual, at least in the short‐term (Rodda et al. 1988; Langkilde and Shine 2006). Such impacts are undesirable, particularly for threatened or rare species (Bradfield 2004), but also when the goal of research is to observe natural population processes and behavior with minimal interference.

Photographic identification has become a popular, non‐invasive alternative for recognizing individuals from natural variation in their markings. The technique has typically been used for mark‐recapture studies which assume that a species displays sufficient phenotypic variation to distinguish among conspecific individuals, that their unique markings are constant through time, and that the markings can be recognized from photographs taken under different conditions (Pennycuick 1978; Bolger et al. 2012). Naturally variable phenotypic patterns on a wide range of taxa, from large mammals (Van Tienhoven et al. 2007; Anderson et al. 2010) to crustaceans (Frisch and Hobbs 2007), have been used for photographic identification of both free‐ranging and captured animals.

In photographic mark‐recapture, individuals are cross‐matched in a library of photo capture histories. However, the time‐expense of manually comparing photo pairs increases exponentially with sample size (Speed et al. 2007; Van Tienhoven et al. 2007; Bolger et al. 2012). One way to overcome the difficulty of cross‐matching large datasets is by computer‐assisted matching of photos of unknown individuals to a reference library. A number of algorithms have been developed for this purpose, but many are highly specialized for particular species or for specific morphological features (Speed et al. 2007; Bolger et al. 2012; Town et al. 2013; Drechsler et al. 2015). A simple and freely available software package, Interactive Individual Identification System, I^3^S Pattern v.4.0.2 (Hartog and Reijns 2014), is a pattern‐matching algorithm that has the potential to be applied to any species with variable markings (Speed et al. 2007; Hartog and Reijns 2014).

Computer‐assisted matching has often been used with large‐bodied free‐ranging marine mammals, where underwater views of the animal is usually unobstructed and evenly illuminated (Speed et al. 2007; Van Tienhoven et al. 2007; Hartog and Reijns 2014). However, even in these conditions, parallax effects of taking photographs at wide horizontal angles (>30°) to the subject can still be problematic for the automated matching process (Speed et al. 2007; Hartog and Reijns 2014). The greater the horizontal angle of deviation from 0° (perpendicular to the subject), the higher the likelihood of a low scoring match (Speed et al. 2007; Rocha et al. 2013).

For smaller taxa (<500 g), parallax effects are likely to be exacerbated because of the comparatively small body areas being photographed. Most studies of smaller‐sized fauna have controlled for the parallax problem by capturing the animal and manipulating it into a fixed position relative to the camera, photographing either in‐hand or using a holding pen (Bradfield 2004; Frisch and Hobbs 2007; Sacchi et al. 2007; Hachtel et al. 2009; Kenyon et al. 2009; Knox et al. 2013; Rocha et al. 2013; Drechsler et al. 2015). This reintroduces the potential stress that the noninvasive technique is supposed to avoid, and involves a large effort to capture the animal for photography.

Because of the often inconspicuous or flighty nature of many herpetofauna, photo‐identification has seldom been applied to free‐ranging individuals of this group. One study showed photo‐identification could be used to track movements of free‐ranging eastern water dragon *Intellagama leseureuii* and calculate their home ranges (Gardiner et al. 2014). However, few reports have investigated the broader limitations of the technique or evaluated alternative ways of using the technique for a free‐ranging reptile.

In this study, we explored the use of photographic identification for a free‐ranging small vertebrate, an Australian skink in the *Egernia* group, Slater's skink *Liopholis slateri* (mean snout‐to‐vent length (SVL) 85 mm). The natural history of Slater's skink, and its varied scale markings (see below), make it a potentially suitable candidate for photo‐identification. We assessed whether unique facial markings of Slater's skink can be used as a reliable means of distinguishing individuals from photographs using (1) an identification key or (2) the I^3^S Pattern algorithm, and whether we could detect any temporal changes in these markings.

## Methods

### Study species

Slater's skink is a rare and globally endangered lizard that exists in small isolated populations within the MacDonnell Ranges bioregion of Central Australia, where it occupies burrow systems located in river floodplains (Pavey 2004). The skink is a diurnal sit‐and‐wait forager, typically spending much of its active time sitting at, or close to, a burrow entrance to bask and ambush passing invertebrate prey (Pavey et al. 2010; Fenner et al. 2012; McKinney et al. 2015). Individuals are easy to observe at these times from as close as 5 m from the burrow, but are difficult to catch without destroying their burrows into which they retreat when more closely approached. Of special relevance is that local population sizes are relatively small (Pavey et al. 2010), allowing the potential for reliable identification among resident individuals, and recognition of any new recruits into the population. Like several other species in the *Egernia* group, individuals have variable spots and facial markings which potentially could be used as unique natural markers (Pavey et al. 2010).

### Study site

Our study site was at Orange Creek, south west of Alice Springs in Central Australia (23°59′S, 133°37′E). At this site a population of Slater's skink occupies a 500 × 200 m area of *Eremophila* shrubland on an alluvial flat. The lizards occupy burrows in soil pedestals that have formed at the base of shrubs by wind and water processes. Over four spring‐summer seasons we detected 115 burrows at the site with evidence of lizard occupation at some time during the period. No other burrows were detected within 5 km of the study site, allowing us to assume we had surveyed an entire population within our site.

### Population survey – photographic mark recapture (PMR)

Over four spring‐summer periods, from December 2011 to April 2015, we photographed all detected individuals, during site visits, usually twice a week. At each visit, one of us (CET) scanned all entrances of each burrow with binoculars (Zeiss 10 × 40) from a distance of greater than 15 m from the burrow entrance. When a lizard was observed out of its burrow, or at the burrow entrance, we photographed it several times (a photographic capture) with a DSLR camera (Canon EOS 450D) and telephoto lens (Canon 70–300 mm). By moving slowly and quietly, we could normally approach to within 4 or 5 m without disturbing the lizard and we attempted to get lateral head photographs from both the left and right side. Each photographic capture was stored in a photo catalogue with assigned information about burrow location.

### Spot development and stability

We documented ontogenic changes in facial markings in three ways. First, we compared the number of spots on temporal, subralabial, and infralabial scales (see below) on six neonates at the end of a summer (early April when young are about 3–4 months old), with the patterns on 29 adult lizards photographically sampled at the same time of year. We assumed spot patterns on left and right sides were related and selected one side (right) to compare spot numbers of neonates and adults using *t*‐tests, and Cohen's D index to evaluate the magnitude of effect size (Cohen 1988). Second, we inspected a 4 month summer time‐sequence of repeated photos of six neonates first observed in December 2012 or in December 2014. We deduced they were the same individuals if they were repeatedly observed as the only juvenile lizard in the same burrow from December to March. Third, we examined photographs for longer term changes (>12 months) in facial patterning in each of the 10 adult individuals that we were able to follow for the entire 4‐year duration of the study. For these 10 mature adult lizards, other distinguishing features such as size, scale shape and arrangement, scars, and other markings, allowed us to be confident that photo sequences were of the same individual.

### Developing a key

**Figure 1:**
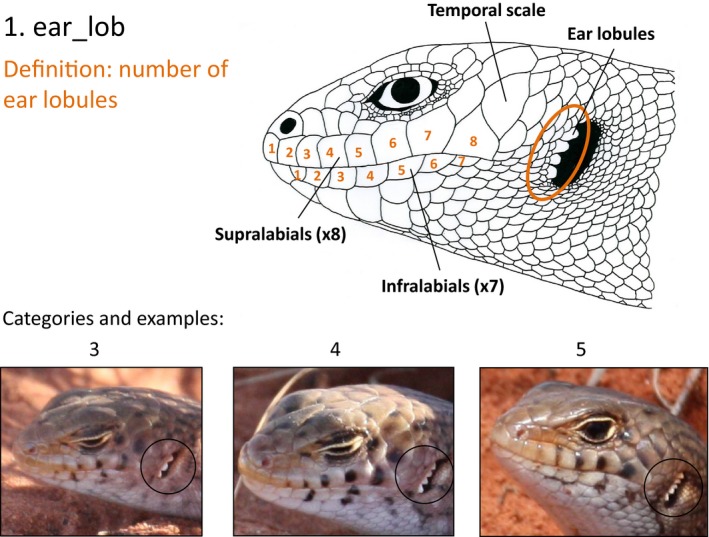
Information provided a priori to observers to enable identification of character 1, ear lobules (Table 1). Similar diagrams were presented for each of the 11 characters.

We used high quality images of 12 adults in the first spring‐summer period to identify characteristics suitable for distinguishing individuals. We targeted the head region, as this is often the most exposed and most easily photographed body part, and within that region we examined ear lobules, melanic spots and scale patterns. In our initial inspection we found these characteristics differed between the left and right sides of an individual lizard. Among left and right profiles of the 12 lizards, we identified 11 characters, each with 2–3 alternative states, which might be used to differentiate among lizards (Fig. 1). We then scored the frequency of each character state for a larger sample of 30 lizards (Table 1).

**Table 1:** The 11 characters used to distinguish individuals of Slater's skink, and frequencies of alternate character states, from 30 individuals

Character		Value	Frequency	Description
1	Number of ear lobules	3	0.04	
		4	0.75	
		5	0.21	
2	Temporal scale marks	0	0.07	Number of discrete, dark markings on the largest temporal scale
		1	0.82	
		2	0.11	
3	Temporal scale marks	0	0.43	Discrete, dark markings on the largest temporal scale touch (1) or do not touch (0) the scale's edge
		1	0.57	
4	Supralabial scales	3	0.64	Number of discrete, dark markings on any of the eight supralabial scales
		4	0.27	
		5	0.09	
5	Infralabial scales	0	1.00	Presence (1) or absence (0) of discrete, dark markings on each of the seven infralabial scales
		1	0.00	
6		0	0.96	
		1	0.04	
7		0	0.66	
		1	0.34	
8		0	0.36	
		1	0.64	
9		0	0.55	
		1	0.45	
10		0	0.46	
		1	0.54	
11		0	0.88	
		1	0.13	

Using the selected characters, we developed an interactive, multi‐choice key with character scores derived for the right and left sides for each of the 30 individual lizards. The key enables the user to select assessed character states in a spreadsheet, for comparison with a library of the previously scored individuals. As each character is scored, the key filters out known individuals in the population that do not display that character state. The user continues to select character states, in any order, either until the spreadsheet identifies a single individual, or until all 11 characters have been scored.

### Testing the key

When testing started after the 2012–2013 spring‐summer season, the photo database contained 1153 images from 314 photo‐captures (mean 3.67 images per capture) of what we considered to be 30 different adult lizards. Our matching of individuals to images was based not only on the character key, but also on other distinguishing features discussed above, and on the tendency of individual lizards to remain at the same burrow for extended periods of time. With continued exposure to the population we came to recognize individuals, but our question was whether we could develop a key that would allow others to identify individuals without that extended experience.

**Figure 2:**
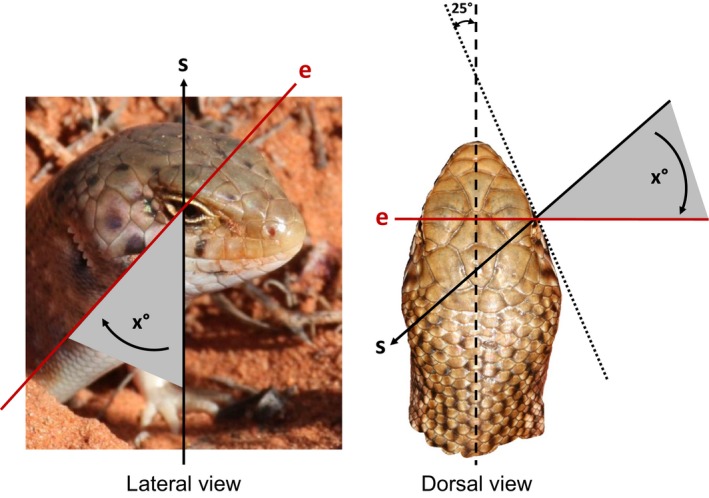
Size of image viewing angle (x°) was estimated by measuring the angle between the line of sight (s) and the line through the center of the eyes (e), and then corrected (−25°) for head tapering; the facial plane (dotted line) tapers at an approximate angle of 25° from the mid‐line of the body (dashed line).

We predicted that images of skinks that were highly angled (>30°) or that had one or more key characters obscured would be more difficult for observers to identify. We tested this by classifying each image into one of three categories according to image viewing angle and the degree to which the key characters were obscured (Table 2). To determine image category, we estimated size of image viewing angle by measuring the angle between the line of sight and the line through the center of the eyes (Fig. 2) using Screen Protractor^™^ software. Because facial profiles were not parallel to the mid‐line of the body but tapered to the snout, we adjusted each measurement by subtracting 25° (Fig. 2). We then randomly selected eight photos from each of the three image categories. The 24 photos were of 14 different individuals with four individuals represented twice and three individuals three times. Our test sample included nearly half of the known population, with some individuals represented by two or three photographs taken at different times and in different conditions. Our sample size was intended to reflect a typical survey period, without imposing too high a load on the volunteer observers. The photos were uploaded to a free online survey tool with response options in a multiple choice format.

**Table 2:** The three photo categories used for testing an identification key for individuals of Slater's skink

Category		Description
1	Full lateral image	Head profile at, or close to, right angles to camera (i.e. angle ≤30°). All characters visible
2	Angled	Head profile at angle to camera (i.e. angle >30°). All characters visible
3	Obscured characters	Characters partially obscured by vegetation/soil/scarring. Head profile at, or close to, right angles to camera (angle ≤30°)

We then asked 24 observers to use the key developed from the previous library to identify the lizards in each of the 24 test photos. We considered that previous experience working with wildlife might improve identification skills in these observers. To test this we selected 12 observers with experience in wildlife survey, and 12 observers with no experience, a sample size that we thought would be sufficient to detect any effect of previous experience. Comparable studies that included a human identification component used a range of three (Frisch and Hobbs 2007) to eight (Knox et al. 2013) observers (mean 5.6; *n* = 3 studies) with varying levels of experience. Each of our 12 experienced observers was a professional field biologist who specialized in plant or animal surveys, although none had specific experience with the study species. None of our 12 inexperienced observers had any advanced training in biology, or professional association with field biology.

The observers were given a 10 minute explanation with examples of each character state (Fig. 1), and then worked independently and with no time limit. We allowed observers to select up to three responses if they were unable to narrow the field to a single candidate individual, since, in practice, the key is not always the ultimate identification step, but often the means to selecting a final few for photo‐comparison. Responses were scored as either correct, if the correct individual was among the selection, or incorrect, for the wrong identification. Observers' test times were recorded by the survey tool, and average times for the two observer types compared with a Kolmogorov–Smirnov test. Times are reported as mean ± SD.

We used a repeated measures ANOVA to examine the effect of observer type (experienced vs. not experienced) and category of photograph (full lateral view vs. angled vs. obscured) on the proportion of correct identifications of the set of photographs. Since both observer types examined the same set of 24 photographs, observer type was a within‐subjects factor, while category of photograph was a between‐subjects factor. To ensure conformity with the assumptions of the analysis the response variable was transformed using an arcsine square root transformation, and effect size calculated using partial eta‐squared (Bakeman 2005). All statistical analyses were computed in R ver. 3.2.1 (R Core Team 2015).

### I^3^S Pattern

The I^3^S Interactive Individual Identification System, originally developed to identify whale sharks (Van Tienhoven et al. 2007), now includes I^3^S Pattern (Hartog and Reijns 2014), which uses photographs of natural body patterns. It calculates a set number of measurements based on differences in patterning after the user has identified three reference points on the photograph and has outlined the region of interest. While the reference points should correct for differences in viewing angle, rotation and scaling, Hartog and Reijns (2014) recommend that images should be taken perpendicular to the line of sight or no more than 30 degrees off that line. The software's key point extraction algorithm generates a ‘fingerprint’ file (a point cloud) for each image which can be compared with other files in the reference library to create a ranked list (Hartog and Reijns 2014). The key points in the fingerprint files are matched for sizes and separation distance to determine potential matching key point pairs. Then a distance metric is calculated by summing the distances between each point pair and dividing by the square of the number of key point pairs (Hartog and Reijns 2014). Lower scores indicate a better match.

**Figure 3:**
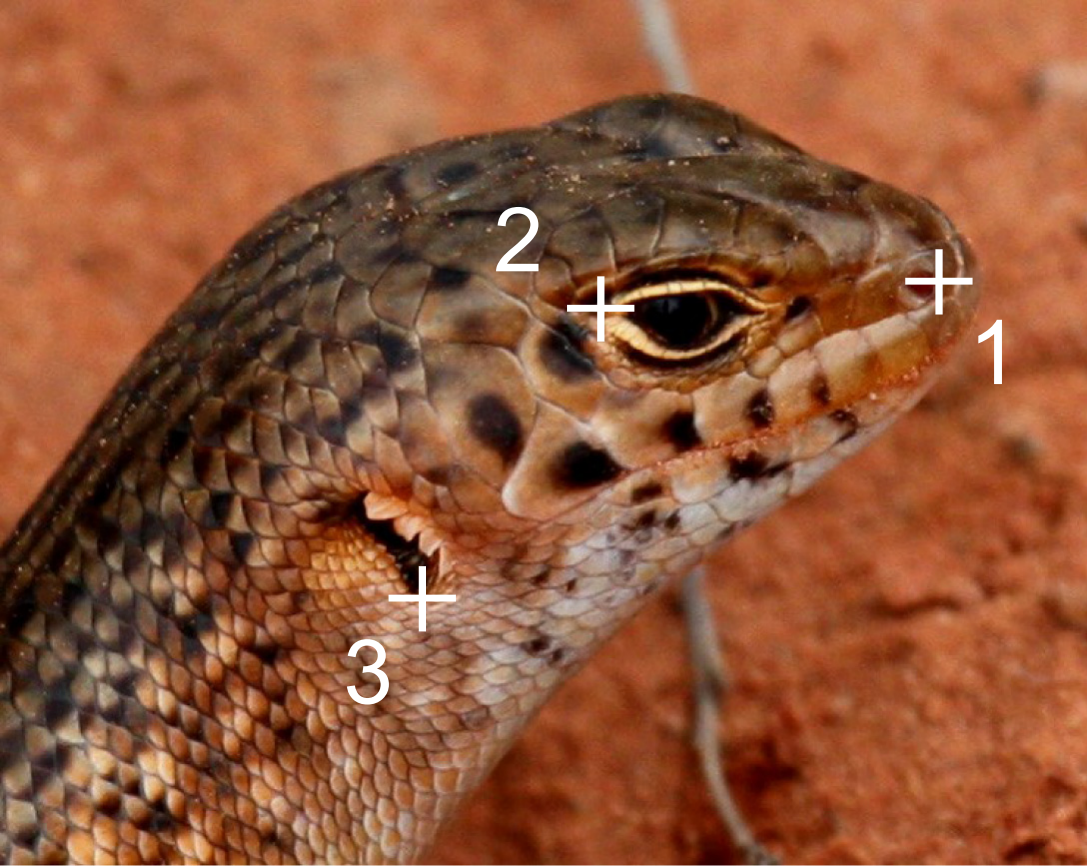
Three reference points selected by the user as required by the I^3^S Pattern software: (1) outer edge of nasal hole, (2) upper corner of eye‐ring, and (3) bottom edge of tympanum.

Where available, we selected three high quality images of the left and right side of each of the 30 individuals used in the key (*n* = 98; for some individuals we only had one or two images per side profile) and loaded them into the I^3^S database. We selected the region of interest to contain 10 of the 11 characters described in the written key; ear lobules were not included. We selected three reference points to form a triangle around the region of interest: outer edge of nasal hole, edge of eye‐ring, and the bottom edge of the tympanum (Fig. 3). Photos were annotated as left or right profile, and fingerprint files were created for each lizard. Then the same 24 test photos that had been used to test the key by independent observers were run through the I^3^S software and matched to the database of the known 30 individuals. We recorded the score metric, rank, and the processing and matching time taken for each test photo.

To get a sense of how well the algorithm could match photos of the same individual with each other, we ran the in‐built simple evaluation test. The entire database of 90 photos was matched with itself, with 94 intra individual comparisons and 8010 comparisons overall. The evaluation test reported the number and percentage of comparisons in the top one to 20 matches (Hartog and Reijns 2014).

## Results

### Spot development and stability

Pigmentation spot patterns in Slater's skink developed during early growth. Right‐side profiles of end‐of‐summer neonates (*n* = 6) had a significantly lower mean spot count than right‐side profiles of all adult skinks at the same time (*n* = 29), on all scored characters: fewer marks on temporal scales (character 2, *t*_(5)_ = 2.74, *P* < 0.05, *d* = 1.65), on supralabial scales (character 4, *t*_(28)_ = 3.82, *P* < 0.001, *d* = 0.77) and on all infralabial scales (sum of characters 5–11, *t*_(13)_ = 4.41, *P* < 0.001, *d* = 1.32). Repeat inspection of juvenile individuals over time showed that these spots appeared and then grew larger and darker over the first summer growth period (Table S1). In multiple images, over periods of 12–36 months, we found 10 mature adults retained identical spot patterns.

### Testing the key

**Figure 4:**
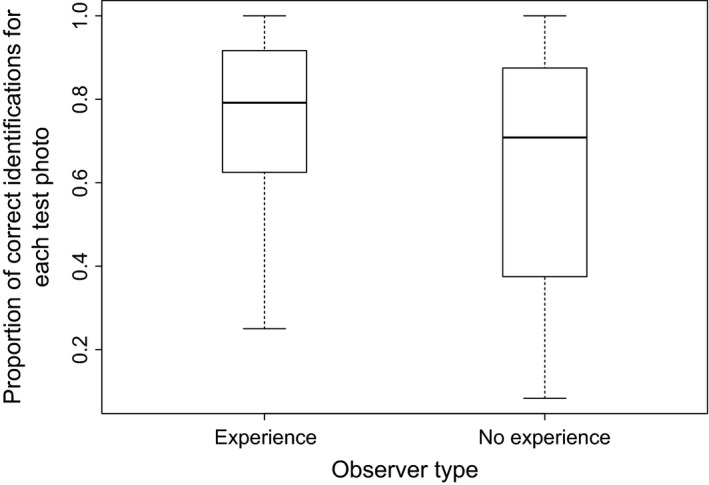
Proportion of correct identifications for each test photo by observers with experience and no experience.

From the 24 test photos, 24 independent observers correctly identified a mean of 16.6 ± 0.77 SE (69%) of individuals. There was no significant effect of category of photograph, nor any interaction effect between category and observer type, but there was a significant main effect of observer type (Table 3). Observers experienced in wildlife survey identified a significantly higher proportion of photos correctly (74%) than observers without experience (64%; Fig. 4, $\eta_{p}^{2}$ = 0.25). There was no significant difference in time taken between observer groups (two‐sample Kolmogorov–Smirnov test *D* = 0.269), with experienced observers taking an average time of 171.8 ± 35.8 s and inexperienced observers 176.5 ± 50.3 s per test image.

**Table 3:** Results of a repeated measures analysis of variance comparing effect of observer group (experience vs. no experience) and category of photograph (full lateral view vs. angled vs. obscured) on the proportion of correct identifications of each test photograph

	*F*	df	*P*
Observer	7.66	1, 21	0.01^a^
Category	0.01	2, 21	0.90
Category × Observer	0.73	1, 21	0.39

a Significant at the 0.05 probability level

We did not quantify the nature of the errors made by the observers. However, for some photos the errors related to a variety of different ‘key choices’ by observers, while in other photos the errors were consistent. Consistent errors appeared to be caused by reflective shine on the subject, poor light exposure, poor focus, or a combination. Observers also appeared to find certain characters more difficult to inspect than others. In particular, markings on each of the infralabial scales (characters 5–11) seemed difficult for observers to distinguish. In one test photo, where the individual skink keyed out without observers needing to make a decision about the infralabial scales, 23 of 24 observers correctly identified the individual.

### I^3^S Pattern

**Figure 5:**
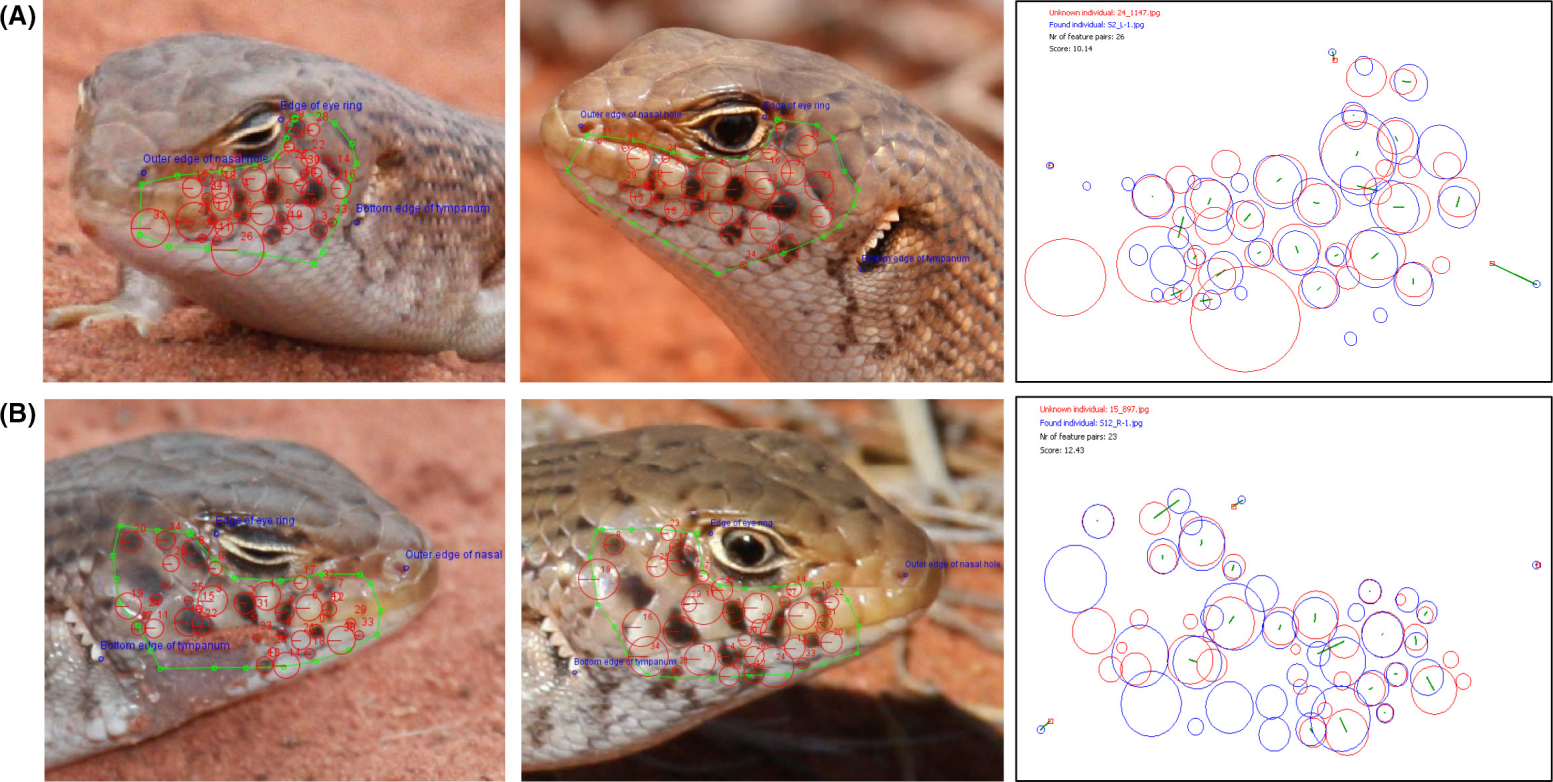
I^3^S Pattern comparisons for matching photographs of Slater's skink. Test photos included (A) subject at extreme angles to camera, and (B) some obscured characters. Diagrams on the right are the corresponding ‘point cloud’ for the two images; green lines indicate distance calculations between matching key point pairs.

The I^3^S Pattern algorithm correctly matched each of the 24 test photos within the top 21 matches. Sixteen (67%) of the 24 test photos were matched as the number one rank, 20 (83%) in the first five ranks, and 22 (92%) in the first 10. Of the eight test photos in each category, I^3^S Pattern correctly matched as the top match, six in category 1 (full lateral image), five in category 2 (angled), and five in category 3 (obscured characters) (Fig. 5). Closer inspection of the two test images for which the correct identity was ranked out of the top 10 choices revealed one of the most widely angled photos (70°) with high image contrast, and the other, a high percentage of vegetation cover over the region of interest. With I^3^S, we could match a test image in an average time of 39.9 ± 47.6 s, plus a processing time of 32.6 ± 4.3 s per image (total time: 73 s or about 40% of the time taken by human observers). The self‐evaluation test calculated 75.5% of correct matches ranked as the number one choice, and 92.7% in the top 20 matches (Table 4).

**Table 4:** Output from self‐evaluation results of the I^3^S software for the database of 56 (30 individuals with unique left and right sides) effective individuals of Slater's skink where the number and percentage of comparisons were calculated in the top #X rank

Rank	Number	Percentage
Top #1	41	74.5
Top #2	44	80.0
Top #3	45	81.8
Top #5	46	83.6
Top #10	48	87.2
Top #20	51	92.7

## Discussion

Our study has been one of the first to explore the use of photographic identification for individuals of a free‐ranging, small terrestrial vertebrate. We showed that with careful examination of facial markings from good quality photos, developing an identification key for individuals is possible in a species that has stable facial markings. We also showed that observers can use the key to score poorer quality photos, whether the face was partly obscured or at wide horizontal angles to the camera. A key that discriminates on characteristics that can be objectively described (e.g. presence/absence of marking on a particular scale) can be used by any observer, regardless of their familiarity with the species, or their experience in wildlife survey. However, the key still requires a subjective assessment by the observer relative to the designer's assessment, and is therefore imperfect. The significantly greater performance by observers with experience suggests that less‐experienced observers could achieve a greater accuracy with more training, time and effort.

Our testing of the computer matching algorithm I^3^S found the identification ability to be no better than human observers. In each photo category, the proportion of correct identifications with automated matching was comparable with that of the human observers'. The software was able to correctly match some individuals from photos that most observers incorrectly identified and vice versa. The software's self‐assessment results showed matching rates below that of photo datasets from other taxa, and accordingly, the developers have concluded that this particular algorithm is not well‐suited to this species (J. Hartog and R. Reijns pers. comm.). We suspect that flash on reflective scales, shadows, variable lighting, and other photo artifacts account for the low self‐matching scores in this dataset. Epidermal shine is common in skinks (Scincidae), as determined by their relatively fine (smooth) microornamentation (Arnold 2002). In comparison, the eastern water dragon's coarse surface structure was not reported to cause reflective issues in photos or be problematic for the I^3^S software (Gardiner et al. 2014). In our study, an insufficient number of high quality reference images likely contributed to the low score in the self evaluation results. Nevertheless, those lower quality images represent a typical sample in our study system. If this automated technique is to be more widely useful it may be that separate new algorithms will need to be developed to account for scale‐shine and other species specific features, or that useable images will need to come from a narrower set of ambient conditions, such as cloudy days.

While automated computer‐assisted identification had a clear time advantage, the higher percentage of correct identifications of experienced observers suggests a possible trade‐off between time and accuracy. If there is some differential rate of misidentification between human and computer assisted techniques, then, particularly for smaller populations, the compromise of taking more time to achieve more reliable identification may be worthwhile.

We have shown that developing an identification key for human observers may be a viable and reliable technique, especially for a finite and small population. Where photographic images can be collected easily, and where there is sufficient variability in marking patterns among individuals, the technique can be used to assess identity without substantial impact on the observed population. While each of the alternative approaches, human or automatic identification has its advantages, it may be possible to use a combination of the two. The key could be used by human observers to narrow the field to a group of individuals that may then be separated based on other behavioral, spatial, or morphological features. In the latter case the computer system may be used.

The photographic key will be particularly valuable in the confident identification of previously recorded individuals, and of new adult entrants into the Slater's skink population, when candidate individuals from the key are combined with additional information from field observations, including spatial stability and other distinguishing features of individual lizards. For this endangered lizard species, the photo‐identification key will be a valuable source of information about spatial structuring of individuals in a population within a season, about social interactions within a population, and about dynamical changes to population numbers across successive seasons. The key will also allow comparable monitoring programs by different personnel in the inevitable case of staff turnover in a conservation management program.

Our technique may have wider direct benefit for camera traps, or motion‐sensor cameras, which are becoming increasingly popular. While, at present, camera traps cannot focus, or target a subject like a human operated camera, they have potential for individual identification of reptile taxa. For example, Welbourne (2013) incidentally observed that he could distinguish individuals of a small agamid lizard, *Amphibolurus muricatus* (body mass <60 g), on the basis of ornamental spots, from camera traps. Recognizing trapped individuals of other species may depend on the resolution of the camera, the size of the animal and the proximity to the camera (Mendoza et al. 2011), and on acquiring multiple images to get the appropriate angle (Hohnen et al. 2013). Our method developed here clearly has potential to be applied to camera‐trapping studies and thus a range of terrestrial wildlife monitoring and management applications.

## Conflict of Interest

The authors have no conflict of interest with any aspect of this study.

## Supporting information


**Table S1**. Temporal sequence of the development of facial markings for an individual of *Liopholis slateri* (S39).
