# Supplementary material for: Photographic identification of individuals of a free‐ranging, small terrestrial vertebrate
Source: Ecol Evol. 2016 Jan 18;6(3):800–9. doi: 10.1002/ece3.1883 (PMC4739561; doi:10.1002/ece3.1883)
Supplement: Supplementary file 1 — Table S1. Temporal sequence of the development of facial markings for an individual of Liopholis slateri (S39). [file ECE3-6-800-s001.docx]

Table S1 Temporal sequence of the development of facial markings for an individual of L. slateri (S39). Spots have been scored according to the characters used in the key (Table 1). Days are days elapsed since first observing the neonate, and observations thereafter. Grey shading denotes row descriptions of the head profile on the right side.

| **days** | **profile** | **ear_lob** | **no_temp_marks** | **temp_touch** | **no_**  **supralab** | **7_**  **infra** | **6_**  **infra** | **5_**  **infra** | **4_**  **infra** | **3_**  **infra** | **2_**  **infra** | **1_**  **infra** | **description** |
| --- | --- | --- | --- | --- | --- | --- | --- | --- | --- | --- | --- | --- | --- |
|  | R | 5 | 0 | 0 | 3 | 0 | 0 | 0 | 0 | 0 | 0 | 0 | three faint spots on supralabial scales |
| 31 | R | 5 | 0 | 0 | 3 | 0 | 0 | 0 | 0 | 0 | 0 | 0 | supralabial spots darker and more defined |
| 31 | L | 4 | 0 | 0 | 3 | 0 | 0 | 0 | 0 | 0 | 0 | 0 | three markings on supralabial scales, faint marking on lower jaw |
| 42 | R | 5 | 0 | 0 | 3 | 0 | 0 | 0 | 0 | 0 | 0 | 0 | faint marks developing on infralabial scales |
| 57 | L | 4 | 0 | 0 | 3 | 0 | 0 | 0 | 0 | 0 | 0 | 0 | spots darker, infralabial outline developing |
| 77 | L | 4 | 0 | 0 | 3 | 0 | 0 | 0 | 0 | 0 | 0 | 0 | spots more defined |
| 105 | L | 4 | 0 | 0 | 3 | 0 | 0 | 0 | 1 | 0 | 0 | 0 | fourth supralabial spot developing |
| 114 | R | 5 | 0 | 0 | 3 | 0 | 0 | 1 | 1 | 0 | 0 | 0 | faint spot developing on temporal scale, infralabial markings darkening |
| 323 | R | 5 | 1 | 1 | 3 | 0 | 0 | 1 | 1 | 0 | 0 | 0 | other markings developing on lower jaw, temporal scale spot darkening |
| 323 | L | 4 | 0 | 0 | 3 | 0 | 1 | 0 | 0 | 0 | 0 | 0 | supralabial markings darker and more defined, temporal scale spot darkening |
| 332 | L | 4 | 0 | 0 | 3 | 0 | 1 | 0 | 0 | 0 | 0 | 0 | other infralabial marks darkening |
| 627 | R | 5 | 1 | 1 | 4 | 0 | 0 | 1 | 1 | 1 | 0 | 0 | facial markings darker and more defined |
| 627 | L | 4 | 1 | 1 | 4 | 1 | 1 | 1 | 1 | 1 | 0 | 0 | facial markings darker and more defined, more marks on lower jaw |
| 638 | L | 4 | 1 | 0 | 4 | 1 | 1 | 1 | 1 | 1 | 0 | 0 | no change |
| 638 | R | 5 | 1 | 1 | 4 | 0 | 0 | 1 | 1 | 1 | 0 | 0 | no change |
